# Left Pulmonary Artery Aneurysm: A Post-stenotic Pulmonary Aneurysm Related to Pulmonary Valve Stenosis

**DOI:** 10.7759/cureus.34836

**Published:** 2023-02-10

**Authors:** Pradnya Brijmohan Bhattad, Zeynep Yukselen, Mohit Bhasin, Mazen Roumia

**Affiliations:** 1 Cardiovascular Medicine, Saint Vincent Hospital, UMass Chan Medical School, Worcester, USA; 2 Internal Medicine, Saint Vincent Hospital, Worcester, USA; 3 Cardiology, Innovation Cardiology, Norfolk, USA

**Keywords:** dilating vascular disease, ct pulmonary angiography, adult congenital heart disease (achd), pulmonic valve stenosis, pulmonary artery aneurysm

## Abstract

Aneurysms of the pulmonary artery are uncommon vascular pathologies that are associated with congenital structural cardiac anomalies, pulmonary hypertension, vasculitis, neoplasm, iatrogenic, and infection. PAAs are commonly asymptomatic and accidentally diagnosed, however, if symptomatic, clinical features are generally non-specific and depend on the etiology of PAA. CT pulmonary angiography remains the gold standard imaging modality and other diagnostic imaging tests include transthoracic echocardiography and right heart catheterization. Definitive treatment of PAA is surgery, however, conservative management with close monitoring should be practiced in patients with poor surgical candidates or surgery is unlikely to improve survival. Here, we report a case of pulmonary artery aneurysm secondary to congenital pulmonary valve stenosis as well as a brief review of the literature regarding pulmonary artery aneurysms.

## Introduction

Pulmonary artery aneurysms (PAAs) are rare vascular pathologies compared to aortic aneurysms, the estimated incidence of being one in 13,696 necropsies according to a study of 109,571 autopsies published by Mayo clinic [1]. PAAs are classified as true or pseudoaneurysms. A true PAA results from the dilatation of all three layers of vascular walls (intima, media, and adventitia) whereas pseudoaneurysms only involve tunica media and adventitia of the pulmonary artery. Pulmonary artery pseudoaneurysms (PAPs) are uncommon but potentially lethal compared to true PAA as pseudoaneurysms have a higher risk of rupture than true aneurysms [2].

PAAs can be proximal (or central) or peripheral based on the anatomic locations. While proximal PAAs arise from the main pulmonary artery, peripheral PAAs arise from segmental or subsegmental arteries. PAA is defined by a dilation of the pulmonary artery to at least 1.5 times the normal diameter, involving all three layers of the vessel wall [2], and is highly associated with pulmonary artery hypertension (PAH). Proximal PAAs are generally asymptomatic unless complications develop from PAH causing rupture, dissection, or sign of compressions [3]. On the other hand, peripheral PAAs are located on an intrapulmonary artery, thus, even unruptured aneurysms can be life-threatening for instance dissection of PAA can be fatal [4].

The diagnosis of a PAA is often an incidental finding on imaging, however, in suspected cases, echocardiography is the first choice. Transthoracic echocardiography (TTE) is an important tool to reveal the presence of proximal saccular PAA but has a limited role in evaluating peripheral PAA. CT pulmonary angiography is the gold standard to evaluate the morphology and the location of PAAs and it can also differentiate from other vascular pathologies, such as pulmonary arteriovenous malformation (AVM) [5].

PAA is classified as acquired and congenital due to its etiology. In this case report, we present a case of PAA secondary to congenital pulmonic valve stenosis status post-pulmonic valve surgical repair and balloon valvuloplasty where careful monitoring and conservative management were practiced.

## Case presentation

A 64-year-old male with a history of coronary artery disease, hypertension, dyslipidemia, known pulmonic stenosis and PAA presented with worsening dyspnea, peripheral edema, and volume overload. He had open pulmonic valve surgical repair at the age of 29 for his congenital heart disease with pulmonic stenosis and subsequent balloon valvuloplasty at age 56. He underwent angioplasty with stent placement in the proximal right coronary artery at the age of 56. His family history was negative for any congenital diseases including congenital heart disease. As he presented with biventricular heart failure with severe right ventricular systolic dysfunction, he underwent further evaluation by a 2D- transthoracic echocardiogram (TTE) and computed tomography angiography (CTA) of the chest with contrast.

A TTE revealed a left ventricular ejection fraction of 40% with a paradoxical septum, increased left ventricular wall thickness with normal left ventricular cavity size, dilated right ventricular size, normal left atrial size, reduced right ventricular global systolic function, dilated right atrium, with a right ventricular systolic pressure 46 mmHg. Valvular pulmonic stenosis with a maximum pressure gradient of 20 mmHg and a mean pressure gradient of 12.8 mmHg with mild pulmonic regurgitation was noted. The dilated and aneurysmal pulmonary artery at 6 cm, pulmonary arterial hypertension, and severe right heart enlargement were notable findings on the TTE.

**Figure 1:**
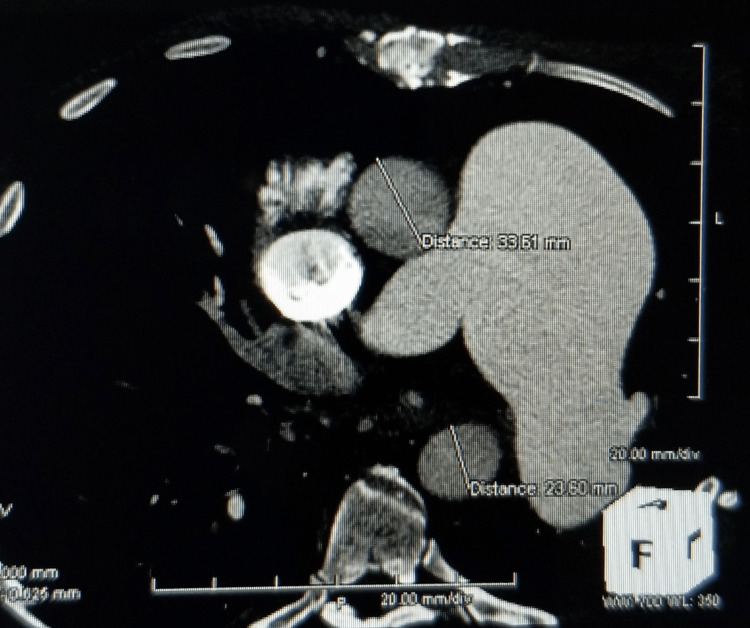
Severe pulmonary artery aneurysmal dilation up to 6.5 cm in diameter

**Figure 2:**
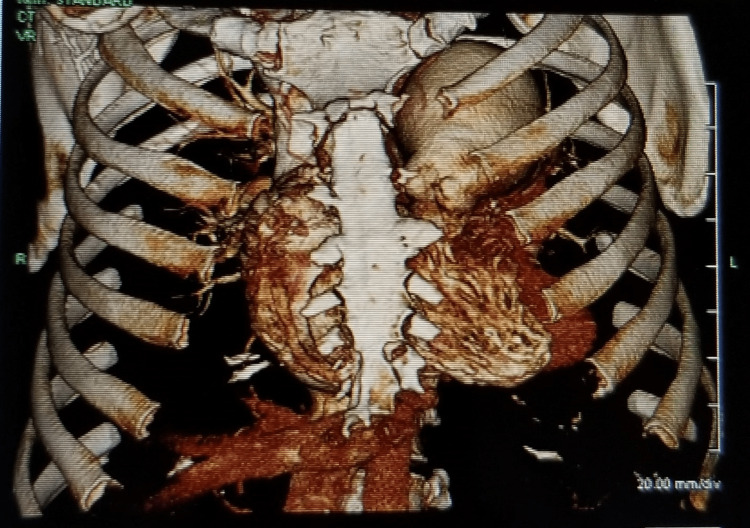
3D reconstruction of CTA showing pulmonary artery aneurysm

**Figure 3:**
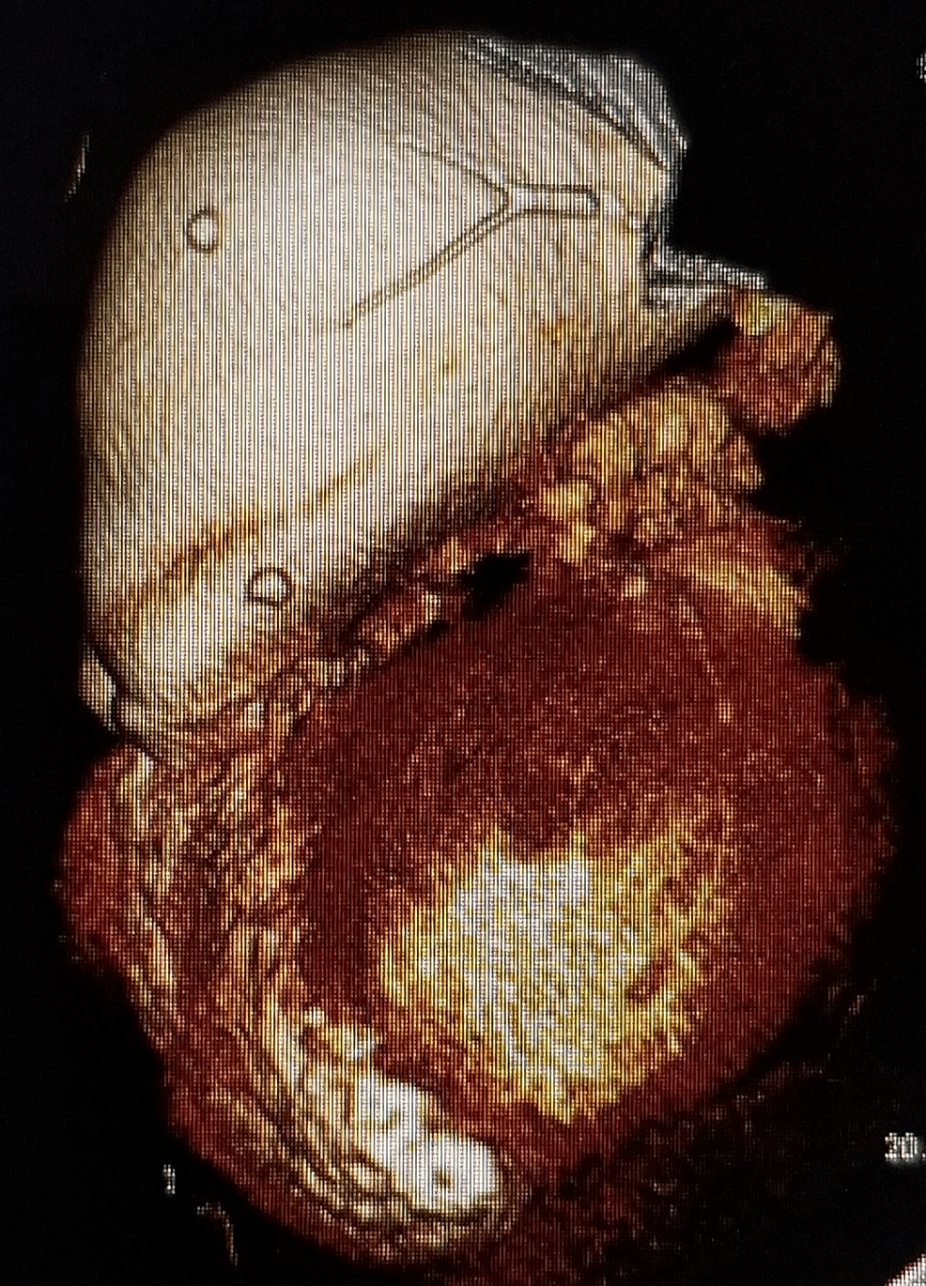
Severe PAA as visualized on 3D CTA

**Figure 4:**
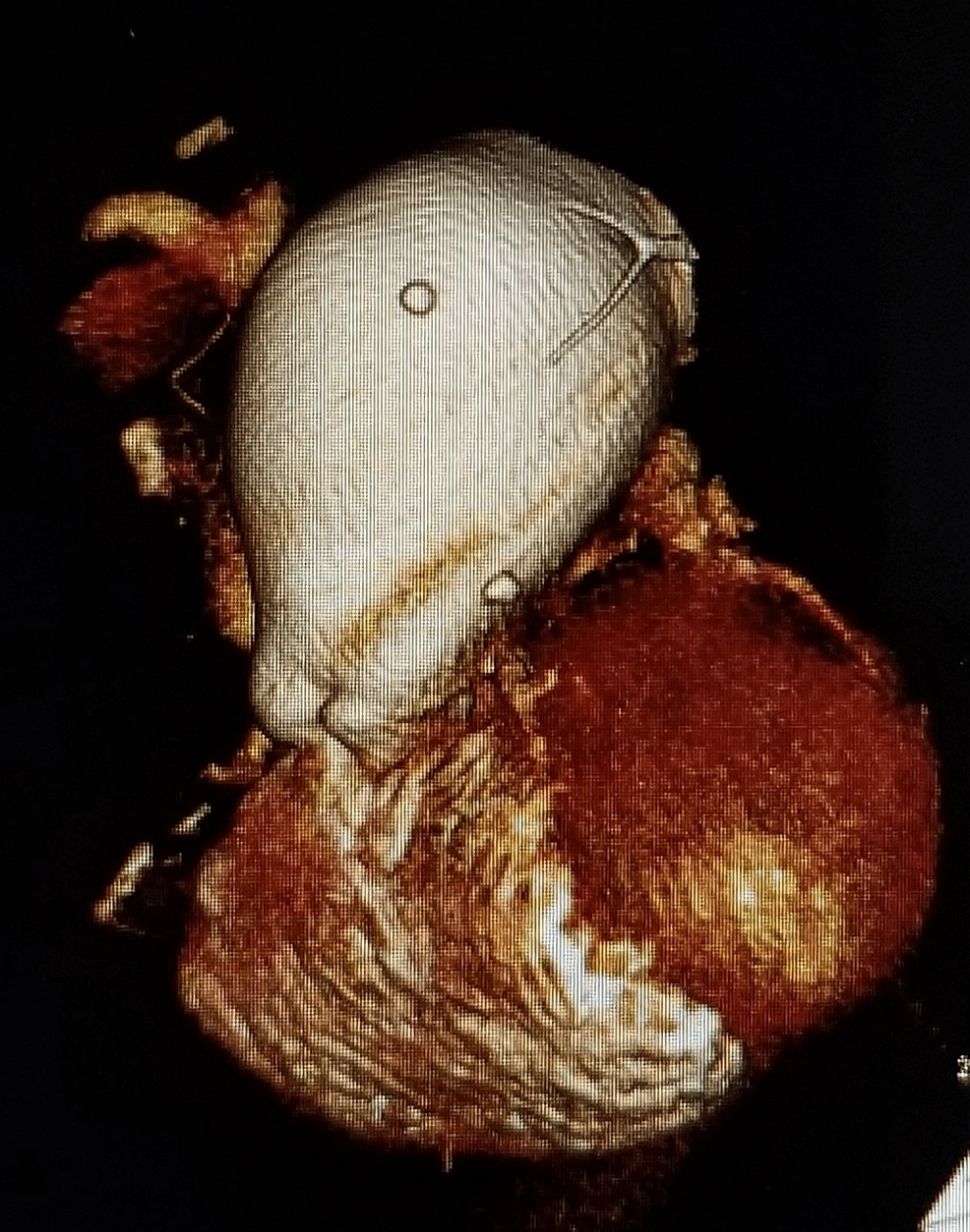
PAA as seen on CTA

**Figure 5:**
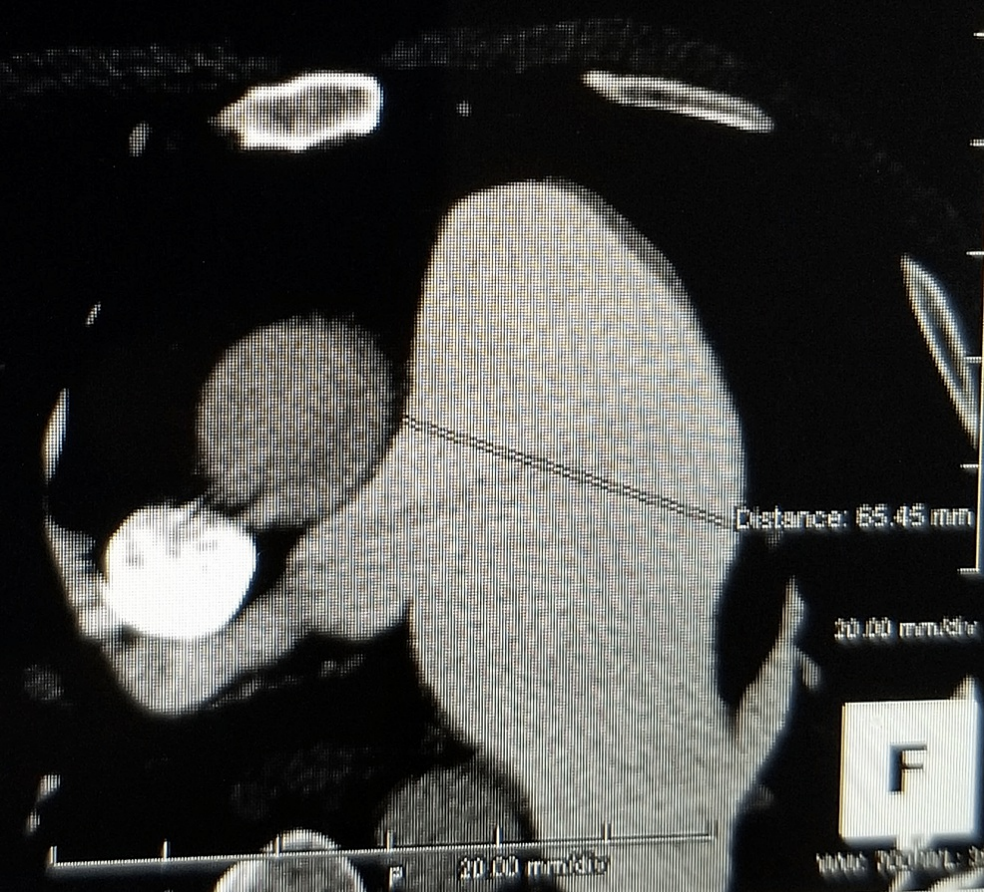
Aneurysmal pulmonary artery as seen on CTA

**Figure 6:**
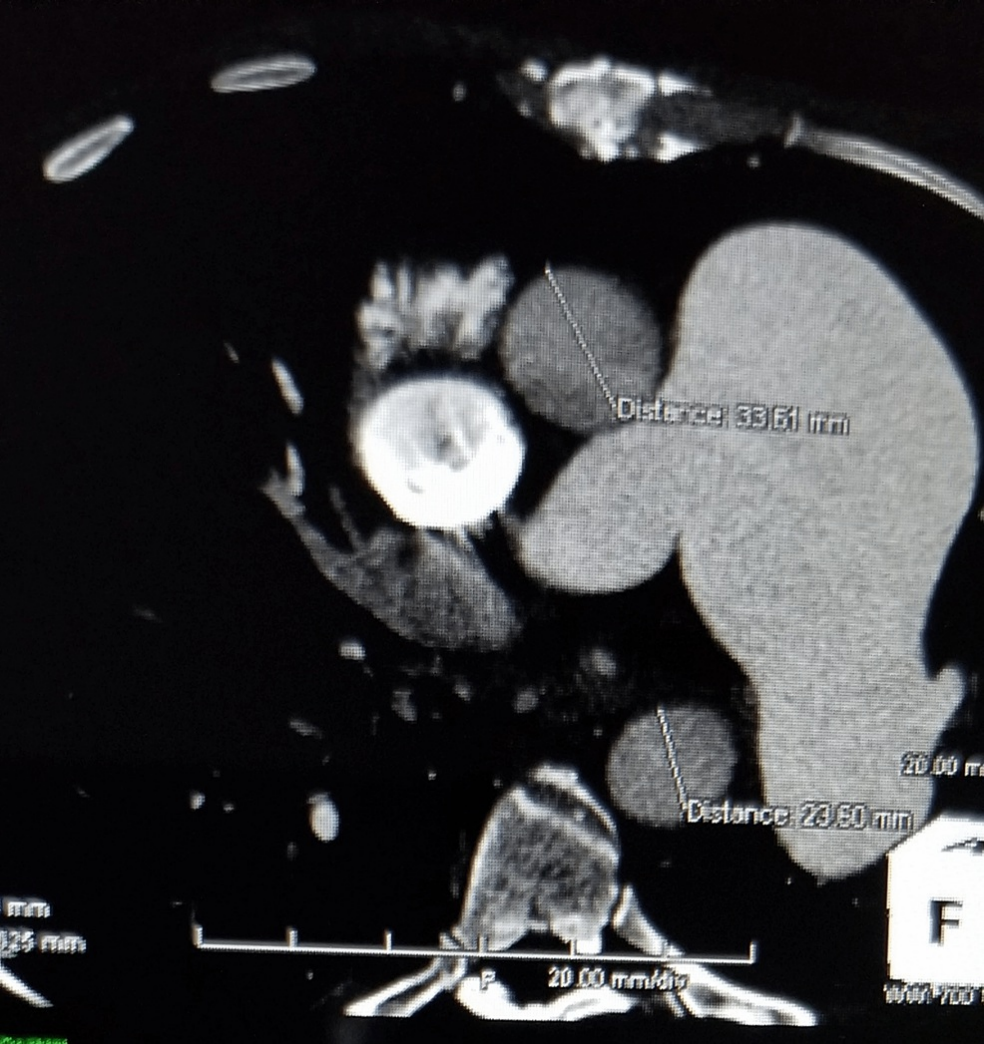
Pulmonary artery aneurysm

**Figure 7:**
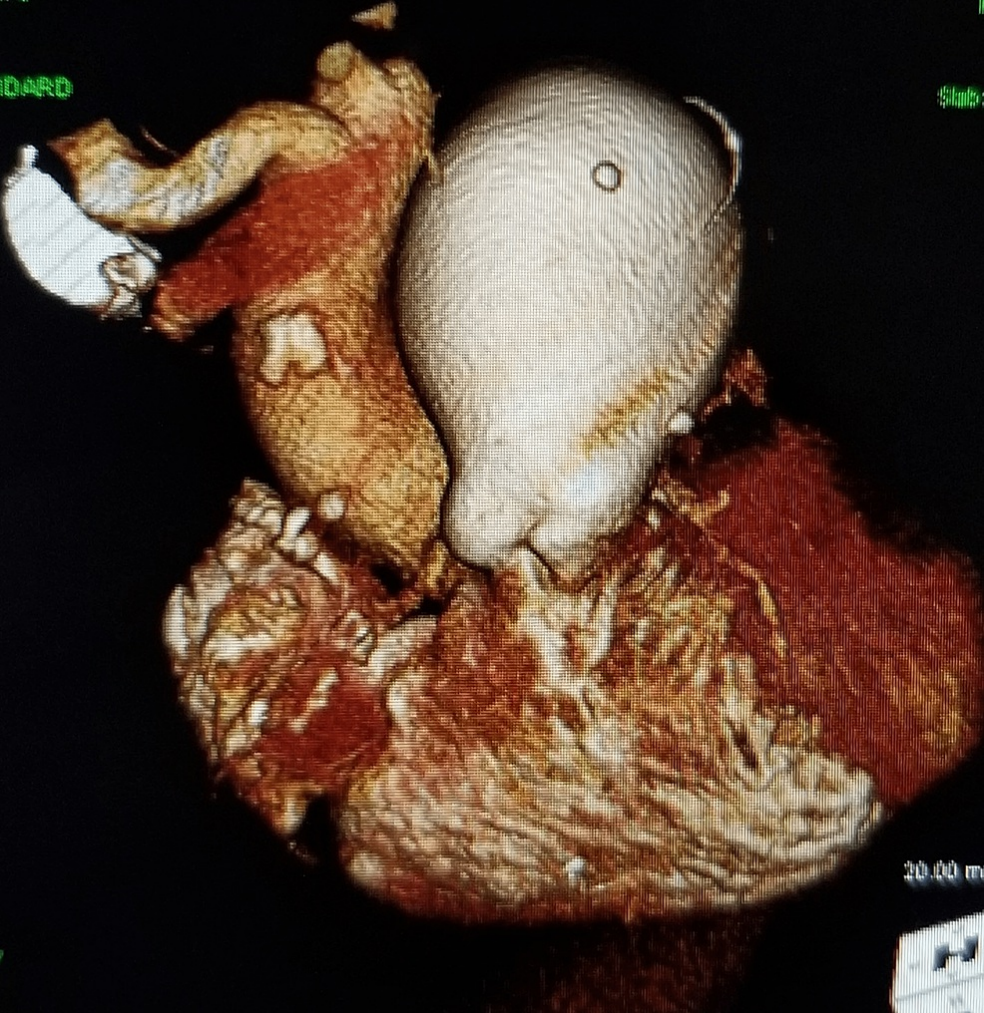
PAA as seen on 3D reconstruction CTA

**Figure 8:**
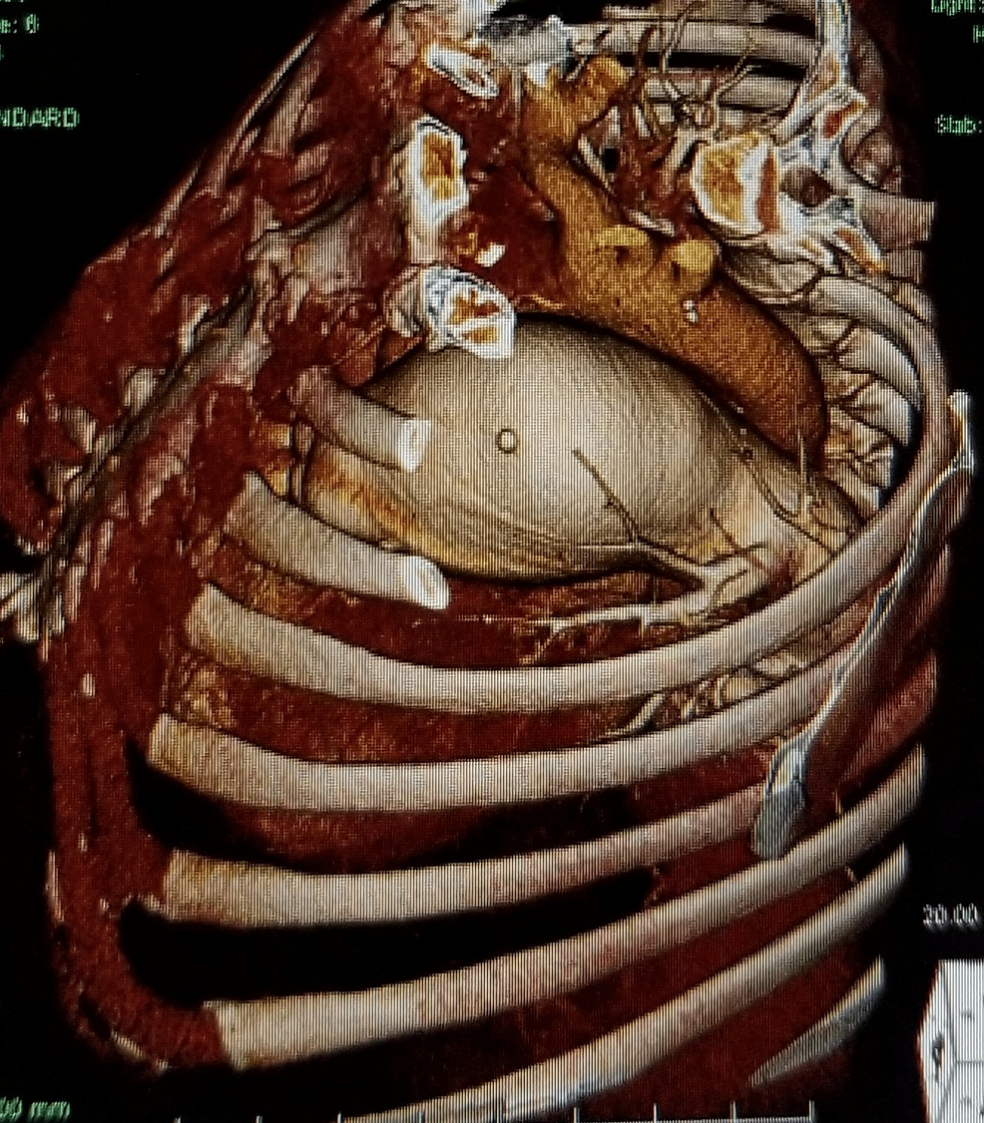
Severe pulmonary artery aneurysm as visualized on CTA with 3D reconstruction

Further evaluation with CTA chest was remarkable for a large 6.5 cm main PAA, enlarged right ventricle, enlarged right atrium with prominent crista terminalis, and a dilated coronary sinus from right atrial hypertension. The pulmonic valve cusps are mildly calcified. A prominent dilated coronary sinus of 16 mm in diameter, secondary to right atrial hypertension. The aortic arch and descending aorta exhibit grade II atheromatous disease, particularly at the ostium of the left subclavian. Also, there is a stent in the proximal right coronary artery with an occluded large ecstatic vessel (Figures 1-8).

Right ventriculography showed a dilated right ventricle with an ejection fraction of 30% with no evidence of infundibular stenosis. There was the visible restriction of pulmonic valve leaflets and a turbulent jet extruding from the right ventricle which filled a massively dilated pulmonary artery. His right heart catheterization revealed mild pulmonic stenosis by a peak gradient of 10 mmHg and a mean gradient of 4 mmHg and moderate pulmonic stenosis by the valve area of 1 cm^2^. He was managed medically given his multiple comorbidities and biventricular heart failure making him a poor surgical candidate for any surgical interventions per multimodal evaluation by cardiology and cardiothoracic surgery.

## Discussion

PAAs can either be acquired or congenital. Common congenital etiologies are structural cardiac abnormalities and connective tissue abnormalities. Historically congenital structural abnormalities causing Eisenmenger’s syndrome that increase blood flow to pulmonary circulation was a common acquired cause of PAA. Gupta et al. reviewed 41 patients with PAA caused by congenital heart abnormalities due to Eisenmenger’s syndrome. The most common cause was patent ductus arteriosus (PDA). Other congenital abnormalities they identified were tetralogy of Fallot (TOF), TOF with absent pulmonary valve, atrial septal defect (ASD), ventricular septal defect (VSD), transposition of great arteries (TGA), and Loeys-Dietz syndrome [6]. Even though rarely published as a case report, pulmonary valve abnormalities are also a significant cause of the congenital etiology of PAAs. In a review shared the outcome of surgical repair of PAA, the most common pathology (>50%) in 38 patients with PAA was pulmonary valve regurgitation (29%), followed by pulmonary valve stenosis (16%), both pulmonary valve stenosis and regurgitation (16%) [7]. In our patient, after excluding mentioned risk factors, congenital pulmonic valve stenosis is likely the leading etiological factor.

Historically, infections such as syphilis and tuberculosis (Rasmussen’s aneurysms) were the primary causes of acquired PAAs, which are less common today. Infective endocarditis is another infectious cause of PAAs that complicates mycotic aneurysms in peripheral pulmonary arteries, which are commonly seen in immunocompromised patients or intravenous drug users. Other acquired PAA pathologies include PAH, vasculitis, neoplasm, (i.e., Hughes-Stovin syndrome Bechet’s syndrome, Takayasu arteritis), trauma, and pregnancy-related or iatrogenic injuries [1,6-9].

In the literature, there are only a few case reports of PAAs secondary to congenital PVS [7,10-12]. Even though the exact mechanism of post-stenotic pulmonary artery dilation is unknown, it was suggested that pulmonary valve stenosis causes PAA due to the turbulent blood flow through the pulmonary artery distal to the stenotic area by weakening of the vessel wall. This phenomenon is also known as the Venturi effect (Bernoulli effect) which refers to an increase in velocity and decrease in pressure when a fluid flows through a constricted section of a tube causing dilation [10]. This effect has been demonstrated in a study to explain the mechanism of the development of aneurysms. In 1954, a study demonstrating the physiology of post-stenotic dilation showed that mild dilation appeared beyond the stenosis after 19 hours of pumping against the rubber tubing with two points of stenosis and dilation subsided when the pumping stopped [13].

PAAs are usually asymptomatic and diagnosed as an incidental finding due to the increased use of CT chest. For symptomatic patients, the clinical manifestations of PAA are usually nonspecific and some of the manifestations include cough, dyspnea, pleuritic chest pain, and hemoptysis [14]. According to a review, among 19 patients with PAAs associated with pulmonary valvular abnormalities, only 11 patients were asymptomatic on the first presentation. In symptomatic patients, the most commonly seen symptom was dyspnea (n=6), followed by exercise intolerance (n=2). Chest pain or angina-like symptoms were also reported in some patients [6]. Similarly, our patient also presented with worsening dyspnea.

Visualization of PAAs on chest x-ray depends on the location and the size of the aneurysm, however, if it appears, it presents as mediastinal or hilar enlargement. If PAA is suspected, CT chest is usually the next step to confirm the diagnosis. TEE should be performed in suspected cases to obtain PA pressures and diagnose PAH [10]. CTA is an important tool for both detection and follow-up of PAAs as it provides the size, location, and characteristics of aneurysms such as saccular or fusiform aneurysm types. Another advantage of CTA is being helpful to reveal the underlying etiology of an aneurysm [15].

Usually, surgery is the preferred treatment for PAA, but a conservative approach should be practiced when the surgery is not feasible due to comorbidities, as seen in our patient. If the patient is asymptomatic without PAH, conservative management can also be practiced [16].

## Conclusions

PAAs are uncommon aneurysms that are usually asymptomatic and diagnosed as incidental findings in imaging tests. CTA remains the choice of imaging modality. Surgery is the definitive treatment; however, management depends on the patient’s symptoms and concomitant comorbidities. We describe the clinical presentation and imaging findings of a patient with a PAA secondary to congenital pulmonic valve stenosis which had been rarely published in the literature. Pulmonary valve stenosis causes pulmonary dilation likely due to turbulent blood flow through the pulmonary artery distal to the stenotic region by weakening of the vessel wall.
